# Update on the role of S100B in traumatic brain injury in pediatric population: a meta-analysis

**DOI:** 10.1007/s00381-024-06565-8

**Published:** 2024-08-23

**Authors:** Alberto Morello, Irene Schiavetti, Enrico Lo Bue, Irene Portonero, Stefano Colonna, Andrea Gatto, Marco Pavanello, Michele Maria Lanotte, Diego Garbossa, Fabio Cofano

**Affiliations:** 1 Neurosurgery Unit, Department of Neuroscience, “Rita Levi Montalcini”, “Città Della Salute E Della Scienza” University Hospital, University of Turin, 10124 Turin, Italy; 2 Department of Health Sciences, University of Genoa, Genoa, Italy; 3 Department of Neurosurgery, IRCCS Istituto Giannina Gaslini, Genoa, Italy; 4 Stereotactic and Functional Neurosurgery Unit, Department of Neuroscience, Rita Levi Montalcini”, AOU Città Della Salute E Della Scienza Di Torino, University Hospital, University of Turin, Turin, Italy

**Keywords:** Traumatic brain injury, Biomarker, S100b, Children, Intracranial injury, Head trauma, Emergency department

## Abstract

**Objective:**

Cranial computed tomography (CT) scan is the most widely used tool to rule out intracranial lesions after pediatric traumatic brain injury (TBI). However, in pediatric population, the radiation exposure can lead to an increased risk of hematological and brain neoplasm. Defined in 2019 National Institute for Health and Care Excellence (NICE) guidelines as “troponins for the brain”, serum biomarkers measurements, particularly S100B, have progressively emerged as a supplementary tool in the management of TBI thanks to their capacity to predict intracranial post-traumatic lesions.

**Methods:**

This systematic review was conducted following the PRISMA protocol (preferred reporting items for systematic reviews and meta-analyses). No chronological limits of study publications were included. Studies reporting data from children with TBI undergoing serum S100B measurement and computed tomography (CT) scans were included.

**Results:**

Of 380 articles screened, 10 studies met the inclusion criteria. Patients admitted with mild-TBI in the Emergency Department (ED) were 1325 (80.25%). The overall pooled sensitivity and specificity were 98% (95% CI, 92–99%) and 45% (95% CI, 29–63%), respectively. The meta-analysis revealed a high negative predictive value (NVP) (99%; 95% CI, 94–100%) and a low positive predictive value (PPV) (41%; 95% CI, 16–79%). Area under the curve (AUC) was 76% (95% CI, 65–85%). The overall pooled negative predictive value (NPV) was 99% (95% CI, 99–100%).

**Conclusions:**

The measurement of serum S100B in the diagnostic workflow of mTBI could help informed decision-making in the ED setting, potentially safely reducing the use of CT scan in the pediatric population. The high sensitivity and excellent negative predictive values look promising and seem to be close to the values found in adults. Despite this, it must be pointed out the high heterogeneity (> 90%) found among studies. In order for S100B to be regularly introduced in the pediatric workflow for TBI, it is important to conduct further studies to obtain cut-off levels based on pediatric reference intervals.

**Supplementary Information:**

The online version contains supplementary material available at 10.1007/s00381-024-06565-8.

## Introduction

Traumatic  brain injury (TBI) is one of the most common encountered pathologies in the emergency department (ED), with an estimated annual incidence of 475.000 cases in children younger than 14 years in the USA [1]. Glasgow Coma Scale (GCS) and Pediatric Glasgow Coma Scale represent reliable clinical tools to rapidly assess the impairment of consciousness level and therefore TBI severity in an emergency setting [2]. In recent years, the incidence of mild TBI (mTBI) has progressively increased [3].

Nonetheless, imaging exams such as cranial computed tomography (CT) are essential to safely rule out potential intracranial complication after TBI [4]. In this context, radiation exposure after cranial CT scans in pediatric population represent a relevant concern due to the increased risk of hematological and brain neoplasm [5, 6]. Consequently, the use of cranial CT scan in children with mTBI must be carefully rationed [7].

Firstly defined in 2019 National Institute for Health and Care Excellence (NICE) guidelines as “troponins for the brain” [8], specific serum biomarkers measurements have progressively emerged as a supplementary tool in the management of TBI due to their capacity to predict intracranial post-traumatic lesions [9]. Among the multitude of biomarkers proposed, S100b is one of the most assessed and it is potentially one of the bio-markers to be evaluated in pediatric TBI cases [10].

In a recent meta-analysis, Oris et al. [11] reported a potential 30% reduction of cranial CT scans for the diagnosis of mTBI when supplemented by serum S100B measurements [11]. Nonetheless, serum S100B concentration is highly variable and strictly depends on the age of the patient, making it difficult to clearly identify a specific serum cut-off [12].

To this purpose, this meta-analysis aims to evaluate the efficacy of serum S100B levels in the identification of intracranial lesion after TBI in pediatric population, defining a potential cut-off to reduce unnecessary cranial CT scan in an emergency setting.

## Materials and methods

### Literature search

This systematic review was conducted following the PRISMA protocol (preferred reporting items for systematic reviews and meta-analyses) [13]. Potentially relevant literature was retrieved from PubMed/MEDLINE, Embase, and the Cochrane Library. The final search was completed on the 21st of December 2023. A detailed search strategy is reported in Supplementary Material 1. Word variations and expanded medical subject headings were searched for whenever feasible.

### Inclusion and exclusion criteria

Articles written in English and involving human subjects were eligible for inclusion. No chronological limits of study publications were included. Prospective and retrospective clinical studies, reports of case series with at least five patients per group and studies enrolling pediatric patients (age < 18 years) presenting to the ED with a history of possible brain injury and undergoing CT scan or inpatient stay, with at least one quantitative blood measurement of S100B on admission and studies that included the possibility of extracting of biomarker sensitivity and specificity were eligible for inclusion. We included studies containing mixed populations; that is, participants with mild, moderate, and severe TBI (according to GCS). We excluded studies using non-quantitative methods to assess biomarker concentrations and studies that analyzed quantitative measurements other than blood (e.g., saliva or urine). Meta-analyses, case reports, or studies with less than five patients per group, cadaver studies, laboratory, and animal studies were excluded.

### Screening and full-text review

Title and abstract screening, full-text review, and data extraction were undertaken in parallel by two reviewers (A.G. and A.M.). Any disagreements at any stage were resolved by discussion and consensus. Persistent disagreements were resolved with the involvement of a third reviewer (E.L.B.). The process was carried out using Rayyan, a tool for undertaking literature and systematic reviews [14].

### Data extraction

The names of the first authors, type of study, publication date, sample size, patient characteristics (age, sex ratio, GCS score), laboratory aspects of S100B (type of assay, concentrations, reference ranges, sampling information, time between TBI and blood sampling), comparison of CT scan versus S100B blood values (negative predictive value (NPV), positive predictive value (PPV), sensitivity, specificity, area under the curve [AUC], and cutoffs), and eventual clinical evolution (CE) were extracted from the studies and collected into a table format.

### Quality assessment

The Cochrane risk-of-bias tool for nonrandomized studies of interventions (ROBINS-I tool) was used for risk-of-bias assessment of the included studies [15]. This was performed by two authors (A.G., and A.M.).

### Statistical analysis

Sensitivity, specificity, NPV, PPV, and AUC were meta-analyzed in R using the Meta and Metafor packages (Version 4.6–0). Missing confidence intervals (CI) were imputed as follows: the lower limit was set as the minimum between sensitivity and 1, while the upper limit was set as the maximum between sensitivity and 0, multiplied by 100. Baseline characteristics were summarized for each study sample and reported as mean (SD) and number (percent) for continuous and categorical variables, respectively, or median (minimum, maximum). The meta-analysis results were visualized using a forest plot with a random effect model. Statistical heterogeneity among studies was assessed by examining forest plots, 95% CIs, and *I*^2^. A likelihood ratio scattergram was plotted to visualize likelihood ratios across studies, facilitating comparison and interpretation of test performance in terms of sensitivity and specificity. Finally, a funnel plot was utilized to investigate potential publication bias. Type I error was fixed at α = 0.05.

## Results

### Literature search

**Fig. 1:**
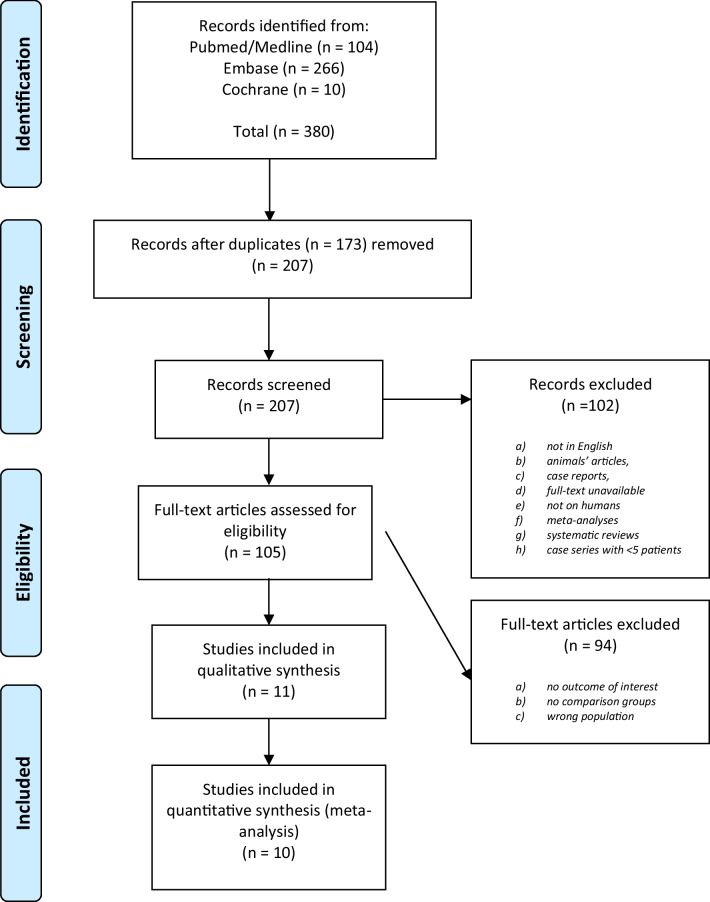
PRISMA flowchart

Our search strategy identified a total of 380 citations. An initial screening to remove duplicate studies produced 207 unique articles. After excluding review articles, experimental studies, meta-analyses, animal studies, and studies with adults, 105 articles were found to be relevant. Finally, 11 studies meeting the inclusion and exclusion criteria, reporting on 1675 patients were identified and included in the qualitative synthesis [16–25], with 10 studies analyzing 1651 patients available for inclusion in the quantitative synthesis (Fig. 1, PRISMA flowchart). The study of Yeung was excluded for quantitative synthesis due to the measurement of a salivary biomarker rather than serum [26].

### Study characteristics and quality

Table 1 provides an overview of the included studies and their quality ratings according to risk-of-bias assessment (ROBINS-I tool). All 10 (100%) studies included in the quantitative analysis were prospective. The majority (80%) of the studies were single-center studies. Patients with mTBI (GCS 15–14-13) at admission to the ED were 1451 (88.47%). In the clinical evaluation at admission, Hallen et al. did not specify the GCS. In most of the cases, S100B serum values were measured within a maximum time-frame of 6 h [16, 17, 21–24]; two authors [19, 20] and just one author [25] recommended a maximum time of 3 and 24 h, respectively. Cutoff values for S100B differed between the studies (concentration threshold 0.006–0.869 µg/L) and researchers interpreted S100B concentrations on the basis of reference ranges adapted for age in only two articles [20, 24]. Cranial CT was the reference examination for all studies included in the quantitative analysis.

**Table 1:** Principle characteristics of the included studies in qualitative synthesis

Study design and cohort details										Diagnostic accuracy for abnormal CT scan									Overall risk of bias
First author, year of publication	Study design	Time of sampling and body fluid	Including poly-trauma	Sample size	GCS 15	GCS 14	GCS 13	GCS < 13	Reference test	Assay test technique	Concentration threshold (µg /L)	S100B, µg/L Mean Median CT + (95% CI)	S100B, µg/L Mean Median CT—(95% CI)	Sensitivity % (95% CI)	Specificity % (95% CI)	PPV (95% CI)	NPV (95% CI)	AUC ROC (95% CI)	
Castellani et al. [16]	Single-center prospective cohort	Serum < 6 h	Yes	109	86	13	10	-	CT	Roche	0.16	0.64	0.50	100 (92–100)	42 (38–43)	0.46 (0.42–0.46)	1.00 (0.90–1.00)	0.68 (0.58– 0.78)	Low
Hallèn et al. [23]	Single-center prospective cohort	Serum < 6 h	Yes	111	-	-	-	-	CT	Roche	0.195	0.28	0.11	100	88	0.32	1.0	0.930 (0.87– 0.99)	Moderate
Bouvier et al. [20]	Single-center prospective cohort	Serum < 3 h	No	446 (n° 424 GCS 15—13)	-	-	-	22	CT	Roche	0.35 (0 –9 months) 0.23 (10–24 months) 0.18 (> 24 months)	0.57	0.28	100 (85–100)	33 (20–50)	0.45	1.00	0.72 (0.59– 0.82)	Low
Babcock et al. [22]	Single-center prospective cohort	Serum < 6 h	Yes	109	83	8	3	15	CT	EIA	0.006	0.210 (SD = 0.313)	0.036	90	31	0.22	0.93	0.71 (0.58—0.80)	Low
Simon-Pimmel et al. [24]	Single-center prospective cohort	Serum < 6 h	Yes	109 (n°109 GCS 15—14)	-	-	-	-	CT	Roche	0.62 (0–3 months) 0.35 (4–9 months) 0.23 (10–24 months) 0.18 (> 24 months)	0.36	0.19	100 (66–100)	32 (23–42)	0.11 (0.05–0.21)	1.0 (0.89–1.0)	-	Low
Manzano et al. [17]	Multi-center prospective cohort	Serum < 6 h	No	73	46	24	3	-	CT	Roche	0.14	0.97	0.35	95 (77–100)	34 (27–36)	-	-	0.73 (0.60– 0.86)	Low
Papa et al. [21]	Multi-center prospective cohort	Serum < 6 h	Yes	114	102	9	1	2	CT	ELISA	0.20	-	-	100 (60–100)	26 (17–37)	-	-	0.67 (0.50– 0.85)	Moderate
Kelmendi et al. [19]	Single-center prospective cohort	Serum < 3 h	No	80	25	26	27	-	CT	Roche	0.105	0.527 (0.447–0.607)	0.145 (0.138–0.152)	100	25.56	-	-	0.893 (0.786–0.987)	Low
Mozafari et al. [18]	Single-center prospective cohort	Serum (not specified)	No	40	17	23	-	-	CT	ELISA	0.172	0.561	0.798	95	100	1.00	0.91	0.985	Moderate
Yeung et al. [34]	Single-center prospective cohort	Saliva < 24 h	No	24 (n° 15 GCS 15–13)	-	-	-	9	CT	EIA	0.869	-	-	76.9	56.0	-	-	0.675	Moderate
Blackwell et al. [25]	Single-center prospective cohort	Serum < 24 h	Yes	425 (n° 284 GCS 15–13)	-	-	-	141	CT	ELISA	-	-	-	48 (0.42–0.54)	51 (0.44–0.59)	-	-	0.51 (0.46–0.56)	Critical

The risk of bias was assessed using the ROBINS-I tool. *n*° = number. *GCS* Glasgow Coma Scale, *EIA* enzyme immunoassay, *AUC* area under the curve, *NPV* negative predictive value, *PPV* positive predictive value, *CT* computed tomography, *CI* confidence interval

Specificity was relatively heterogeneous, in some cases weak (25.56 to 100%). Optimal sensitivity was measured in most cases, but in one study researchers obtained a sensitivity of 48% [25]. Areas under ROC curves ranged between 0.51 and 0.985. PPV and NPV showed relatively heterogeneous results, ranging from 0.11 to 1.0 for PPV and from 0.91 to 1.0 for NPV.

### Quantitative results

**Fig. 2:**
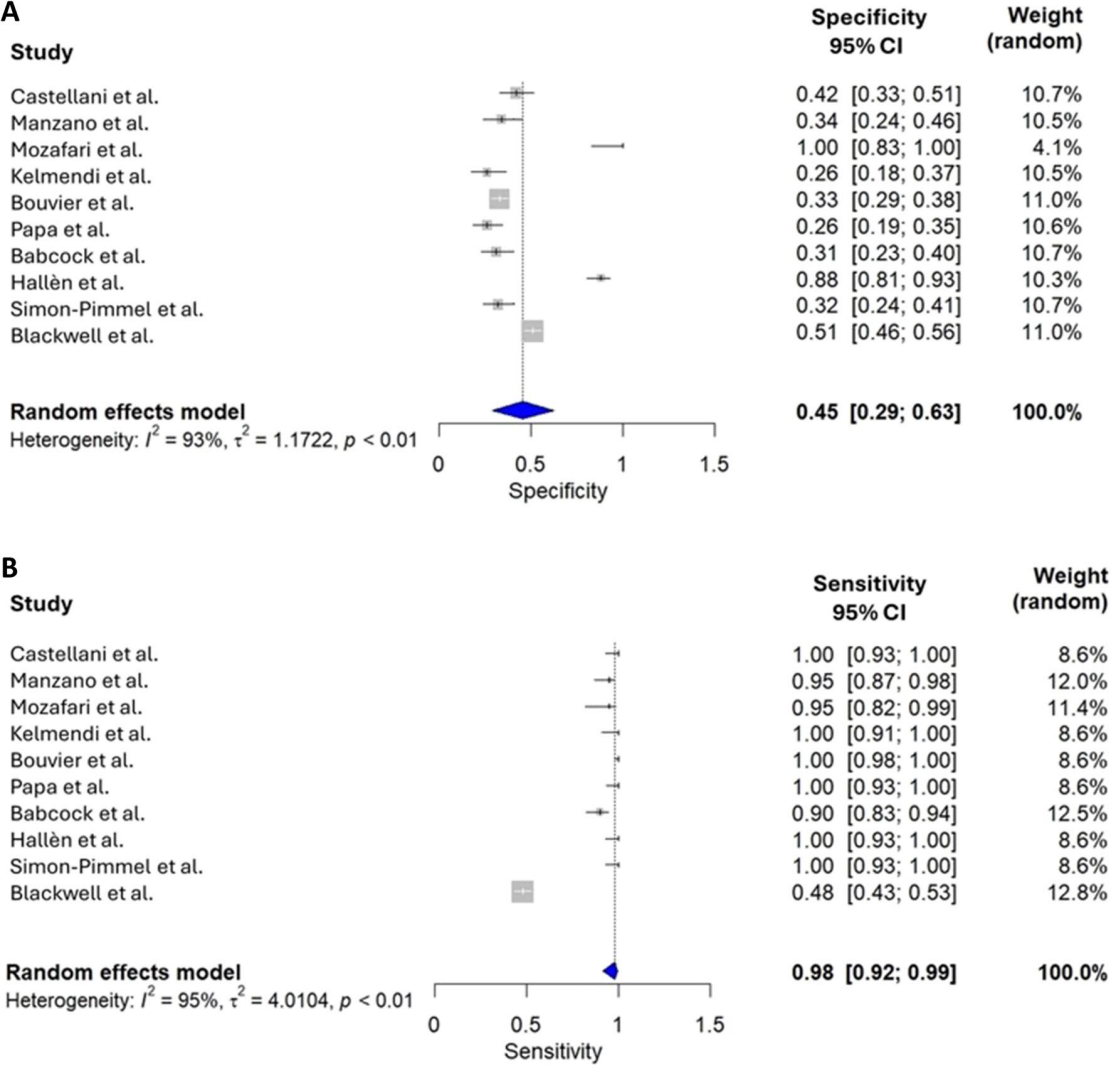
**A** Forest plot showing the individual and pooled specificity of S100B for CT scans (*n* = 10 studies). **B** Forest plot showing the individual and pooled sensitivity of S100B for CT scans (*n* = 10 studies)

**Fig. 3:**
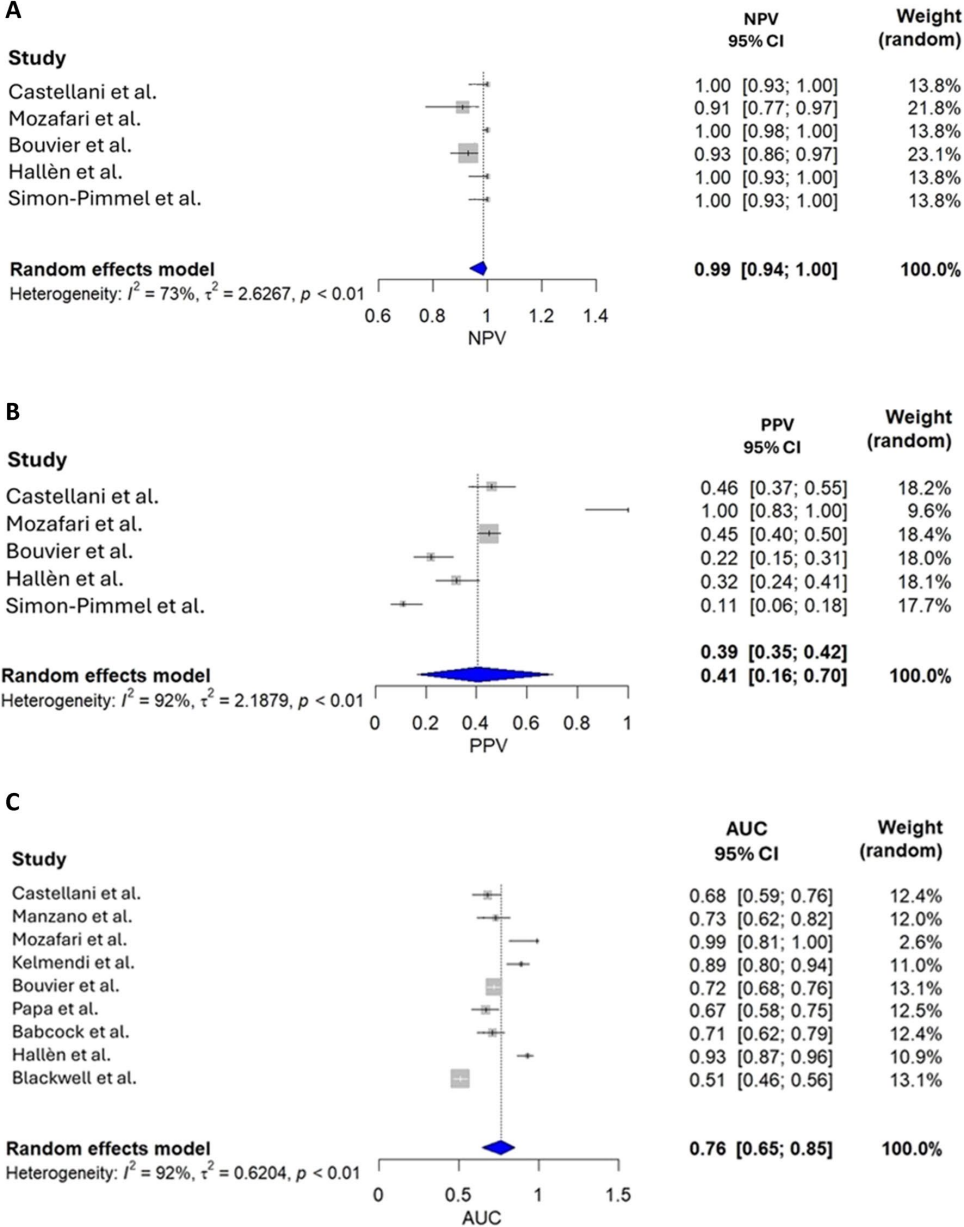
**A** Forest plot showing the individual and pooled NPV of S100B for CT scans (*n* = 10 studies). **B** Forest plot showing the individual and pooled PPV of S100B for CT scans (*n* = 10 studies). **C** Area under the curve (AUC) (*n* = 10 studies)

**Fig. 4:**
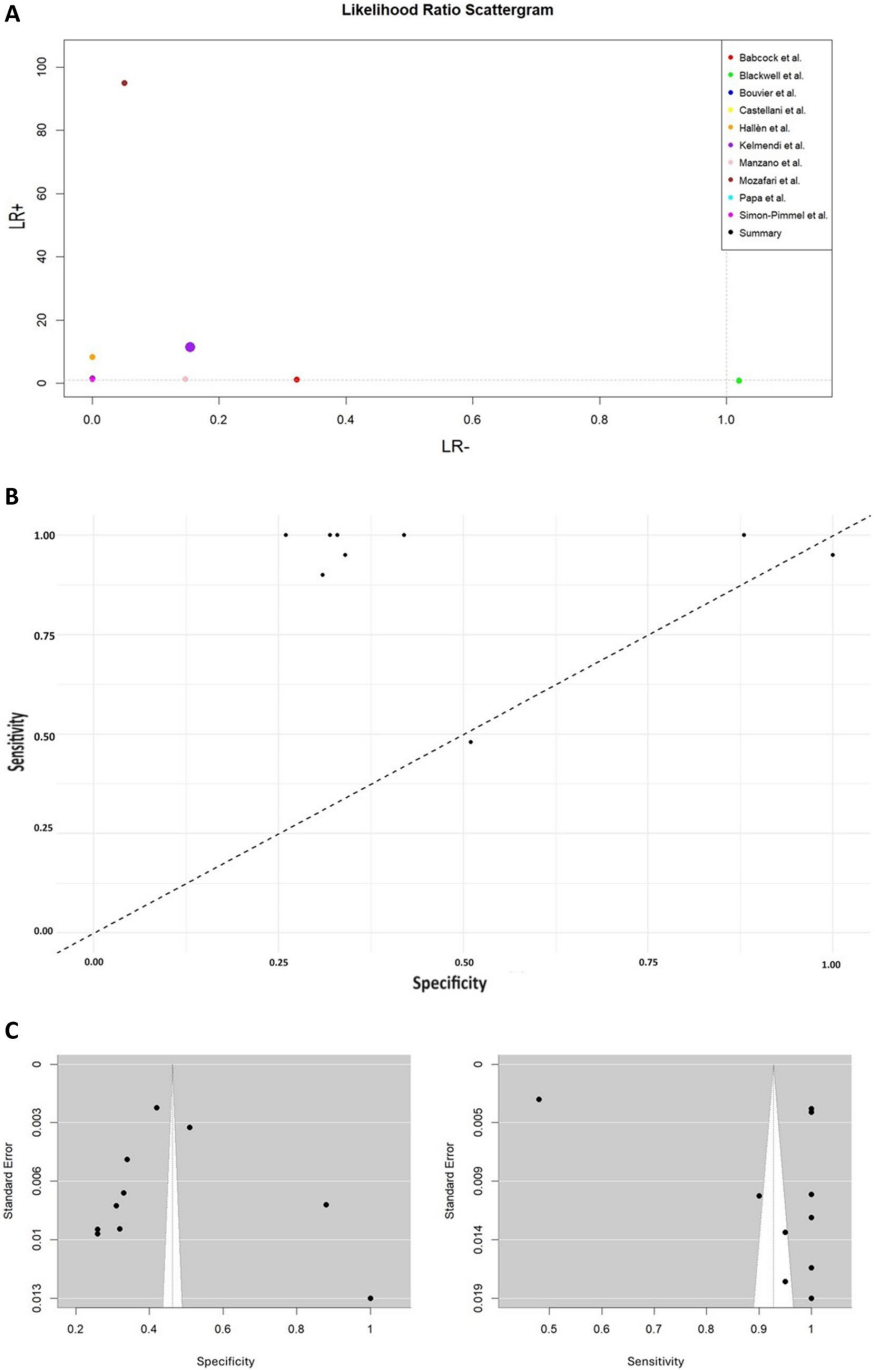
**A** Likelihood ratio scattergram showing individual and pooled LR − and LR + of S100B for CT scan (*n* = 10 studies). **B** Funnel plot screening the distribution of 10 studies. **C** Two funnel plots illustrating sensitivity and specificity on the X-axis against standard error on the Y-axis

After removing Yeung et al. study [26], the meta-analysis revealed a sensitivity of 98% (95% CI, 92–99%) and specificity of 45% (95% CI, 29–63%), resulting in a high NVP (99%; 95% CI, 94–100%) and a low PPV (41%; 95% CI, 16–79%). AUC was 76% (95% CI, 65–85%) (Figs. 2 and 3). There was a significant heterogeneity (> 90%) among the studies for all metrics except the NVP (73%). The likelihood ratio scattergram confirmed the heterogeneity of the studies for both sensitivity and specificity (Fig. 4A).

## Publication bias

Funnel plots of the investigated outcomes can be found in the Fig. 4B, C.

## Discussion

Management of TBI after head trauma in pediatric population represents a challenging situation for the ED clinician. The decision to acquire a cranial CT balances the incidence of detecting an intracranial injury with the risks of unnecessary radiation exposure [7, 27]. Approximately 90% of head CT scan performed for mTBI in pediatric patients did not demonstrate neurological injury [28]. Between 1996 and 2005, the overall use of CT imaging in the ED setting has doubled in children under 5 years and tripled in children between 5 and 14 years, with an estimated 50% increase of head CT [29]. In this context, in the last years, the Pediatric Emergency Care Applied Research Network (PECARN) has produced several studies focused on this specific topic [7]. In 2009, Kuppermann et al. conducted a multicenter prospective cohort study with the largest pediatric population with mTBI, with the primary endpoint to identify patients with very low risk of clinically relevant TBI in which the CT exam could possibly be unnecessary [7]. In 2011, the group of Pandor et al. proposed a methodological algorithm for the management of mTBI in pediatric population [30].

Specifically, Miglioretti et al. in 2017 analyzed the correlation between the lifetime risk of leukemia and a history of previous cranioencephalic CT scans, showing an estimate 1 case per 10,000 CT scans in patients younger than 5 years, and 0.5 cases per 10,000 CT scans in the population between 10 and 14 years [29].

While cranial CT scan remains a standard diagnostic tool, rapid sequence MRI without sedation has progressively been used in the ED, taking advantage of its ability to rule out a wider spectrum of intracranial pathologies compared to conventional CT scan [31, 32]. Nevertheless, the limited availability of MRI compared to CT still represents a major restriction regarding its application in the diagnostic algorithm of TBI in the ED setting.

In this context, the challenges associated with accurate diagnostic and therapeutic workflow for TBI have progressively shed light on the search for blood and fluid biomarkers as potential complementary diagnostic tools, aiming to ultimately optimize neuroimaging management in the ED and improve cost savings for healthcare systems [33]. Despite the promising outlook and multiple research studies, systematic adoption of biomarkers into clinical practice remains limited. Standardization of biomarker assays, validation across heterogeneous patient populations, and systematic integration of biomarkers into existing clinical workflows still represent relevant concerns. Nevertheless, there has been a considerable contribution to the evidence supporting the relevance of biomarkers in TBI diagnostic workflow, with multiple attempts to identify accurate and reliable biomarker suitable for an ED setting. To date, the most evidence for the use of biomarkers as complementary tools for diagnosis and prognosis in pediatric patients with TBI exists for S100 calcium-binding protein B (S100B), glial fibrillary acidic protein (GFAP), ubiquitin C-terminal hydrolase (UCH-L1), and osteopontin (OPN) [25]. Overall, serum and saliva S100B levels have emerged as a sensible and reliable biomarker for brain injury after head trauma in children [23, 34].

S100B is a calcium-binding protein highly expressed by astroglial and neuronal cells. Structurally, is a small dimeric protein with a molecular weight of approximately 21 kDa [20]. It is released from brain cells and enters the systemic circulation probably due to increased permeability of the blood–brain barrier, subsequently being excreted within approximately 6 h from injury [35, 36]. S100B is also expressed in adipocytes, chondrocytes, melanocytes, and epidermal cells to a lesser extent, potentially leading to increased systemic levels even in the absence of brain injury [17], although minor peripheral injuries typically do not cause significant rises in S100B serum levels [37]. Several studies have shown an inverse correlation between S100B serum levels and age, with levels decreasing from infancy to adolescence, particularly during the first 2–3 years of life. This trend can be explained by multiple factors, such as increased permeability of the blood–brain barrier, accelerated protein turnover in neuronal cells, and diminished renal excretion of S100B. All these factors must be considered when analyzing potential reference intervals of serum S100B in a pediatric population, especially during the first years of life [20]. In addition, considering its relatively short half-life ranging from 20 to 120 min, S100B can be used as a biomarker for traumatic brain injury when measured within a timeframe that aligns with its clearance kinetics [16, 20]. Typically, it is most useful for assessing acute injury within 3 h of the traumatic event.

The clinical use of S100B as a biomarker for mTBI was firstly reported in 1995 [38]. Since then, several studies demonstrated its capacity to reduce the number of CT scan conducted in patients with mTBI, especially in adults [39]. Integration of S100B concentrations and clinical decision in adult patients with mTBI can reduce the number of negative CT scan by up to 30% [30, 40]. On this basis, in 2013 the clinical use of S100B in the management of mTBI in adult patients has been firstly introduced in the Scandinavian Guidelines [41].

Nonetheless, the role of S100B in clinical practice for pediatric TBI is still debated. In 2015, another major work was carried out on the Scandinavian Guidelines for the Management of Mild and Moderate Head Trauma in Pediatric Children by Astrand et al. [42]. In the Scandinavian Guidelines for initial management of minor and moderate head trauma in children, Astrand et al. did not include serum S100B evaluation considering the limited number of available studies in the literature, and the high heterogeneity of reference levels related to the age of the patients. In this context, Bouvier et al. demonstrated that serum concentration of S100B in healthy children is higher in the first 3 years of life [43]. As emerges by our analysis, only two studies interpreted serum S100B concentrations references considering also the age of the patients [20, 24].

In this meta-analysis, the high sensitivity and excellent negative predictive values look promising and seem to be close to the values found in adults [36]. Despite this, we must point out the high heterogeneity (> 90%) among the studies for all metrics except the NVP (73%) **(**Fig. 3**).** Although it has high sensitivity, using only serum S100B concentration for initial mTBI screening is not ideal due to the risk of false-negative results. We revealed an important deviation with one study [25]. Finally, the variability of protein concentration by sampling time and particularly by age is an important limitation [44].

In this context, a randomized, multicenter, open-label, prospective, interventional study (nine centers) was recently conducted in France in which researchers used a stepped wedge cluster design with two arms (“S100B management” intervention group and “conventional management” control group) [45]. In this randomized clinical trial including a cohort of 2078 children, S100B biomonitoring produced a reduction in the number of CT scans and in-hospital observation. However, the difference in CT scans performed between the control group and the S100B biomonitoring group was not statistically significant (*P* = 0.44). Another interesting result of this study is that Bouvier et al. found a relative risk of 0.49 (95% CI, 0.30–0.77) in the post hoc analysis for CT scans and 0.46 (95% CI, 0.39–0.51) in the modified intention-to-treat analysis for in-hospital observations.

From the available data, there are no major studies in the literature in which S100B identified specific types of intracranial lesions. In some cases, subdural hematomas have been classified slightly more frequently as false negatives [46, 47]. We speculate that this may be due to the location and/or extent of the brain lesion and the pathoanotomic and neurovascular characteristics of the different lesions that cause altered or delayed leakage of S100B into the circulation. On the other hand, Bouvier et al. found that the S100B identified patients with poor clinical evolution (CE) with a sensitivity of 100% (95% CI, 84–100) and specificity of 36% (95% CI, 31–41) [20]. Specifically, poor CE was defined by the following clinical symptoms: vomiting, facial paralysis, movement disorders, vertigo, photomotor reflex disorders, seizures, progressive headache, or behavioral changes. They showed a significant (*P* = 0.0001) capacity of S100B to differentiate between poor CE and good CE in patients after mTBI and the best threshold conserving a sensitivity of 100% was 0.19 µg/L.

## Conclusion

Despite the undoubtable role of CT imaging, clinical assessment represents a fundamental complementary diagnostic element in the management of pediatric mTBI. The primary objective of this meta-analysis was exploratory and aimed to highlight the possibility of using serum S100B levels in the diagnostic workflow of pediatric TBI. The promising potential of S100B integration in the management of pediatric head injury is evident. Based on this meta-analysis, the measurement of serum S100B could help informed decision-making in the ED setting, potentially safely reducing the use of CT scan in the pediatric population. S100B protein serum levels, in combination with the PECARN algorithm, could ultimately reduce the need for CT scans. The primary goal of this analysis has been to highlight the sufficiency of the evidence in this area, rather than offering specific treatment recommendations. The number of standardized studies is still insufficient, and the variability of protein concentration by age and sampling time should be studied in more detail. In order for S100B to be regularly introduced in the pediatric workflow for TBI, it is important to conduct further studies to obtain cut-off levels based on pediatric reference intervals.

## Supplementary Information

Below is the link to the electronic supplementary material.

Supplementary file1 (DOCX 15 KB)

## Data Availability

Data is provided within the manuscript or supplementary information files.

## Abbreviations

TBI: Traumatic brain injury
GCS: Glasgow Coma Scale
PECARN: Pediatric Emergency Care Applied Research Network
AUC: Area under the curve
CI: Confidence interval
NPV: Negative predictive value
CT: Computed tomography
