# Supplementary material for: Update on the role of S100B in traumatic brain injury in pediatric population: a meta-analysis
Source: Childs Nerv Syst. 2024 Aug 23;40(11):3745–56. doi: 10.1007/s00381-024-06565-8 (PMC11538191; doi:10.1007/s00381-024-06565-8)
Supplement: Supplementary file 1 — Supplementary file1 (DOCX 15 KB) [file 381_2024_6565_MOESM1_ESM.docx]

**Supplementary Content 1 - Detailed Search Strategy**

| **Database** | **Search terms** | **Exceptions** | **Strategy** | **Results** |
| --- | --- | --- | --- | --- |
| Pubmed/MEDLINE | Traumatic brain injury; pediatric; children; S100b; neuron specific enolase | - | "S100B" AND "traumatic brain injury" AND ("pediatric" OR "children")  "neuron specific enolase" AND "traumatic brain injury" AND ("pediatric" OR "children") | 64  40 |
| Embase | Traumatic brain injury; pediatric; children; S100b; neuron specific enolase | - | "S100B" AND "traumatic brain injury" AND ("pediatric" OR "children")  "neuron specific enolase" AND "traumatic brain injury" AND ("pediatric" OR "children") | 174  92 |
| Cochrane Library | Traumatic brain injury; pediatric; children; S100b; neuron specific enolase | - | "S100B" AND "traumatic brain injury" AND ("pediatric" OR "children")  "neuron specific enolase" AND "traumatic brain injury" AND ("pediatric" OR "children") | 6  4 |

**Supplementary Table 1.** Search strategies stratified by databases.
